# Complete genome sequence of *Pseudalkalibacillus* sp. Hm43 isolated from marine sponge *Haliclona caerulea* in the coral reef

**DOI:** 10.1128/mra.00540-26

**Published:** 2026-07-16

**Authors:** Wen-Wu Zhang, Yue Su, Yuan-Yuan Chen, Yu-Chen Deng, Cong Sun, Lin Xu, Rong-Gen Jiang

**Affiliations:** 1 Trend Biotech Co., Ltd., Hangzhou, People’s Republic of China; 2 College of Life Sciences and Medicine, Zhejiang Sci-Tech University 12646, Hangzhou, People’s Republic of China; 3 Zhejiang Engineering Research Center for the Development Technology of Medicinal and Edible Homologous Health Food, Shaoxing Biomedical Research Institute of Zhejiang Sci-Tech University Co., Ltd., Shaoxing, People’s Republic of China; 4 Ministry of Natural Resources, Third Institute of Oceanography, Xiamen, People’s Republic of China; Montana State University, Bozeman, Montana, USA

**Keywords:** complete genome, *Pseudalkalibacillus*, marine sponge, chitin degradation

## Abstract

We report the complete genome sequence of *Pseudalkalibacillus* sp. Hm43 isolated from marine sponge *Haliclona caerulea* in Guangxi coral reef, China. The genome consists of a circular chromosome (3,739,586 bp; guanine-cytosine [GC] content: 43.4%), including three GH18 chitinase genes. Strain Hm43 exhibits exochitinase and endochitinase activities.

## ANNOUNCEMENT

The genus *Pseudalkalibacillus* belonging to the phylum *Bacillota* was established by Joshi et al. in 2021 (1). *Pseudalkalibacillus* strains were isolated from diverse marine environments, but not marine sponges (1–4). Marine sponges contain diverse microorganisms contributing to host health via nutrient provision and waste degradation (5–7). Here, we obtained the first complete genome of a *Pseudalkalibacillus* strain from marine sponge, advancing understanding of host-microbe interactions and microbial resource development.

The marine sponge *Haliclona caerulea* sample was collected at a coral reef around Weizhou Island, China (21.02°N, 109.08°E). After being rinsed with sterile 3.0% NaCl and homogenized, the sample was spread on modified marine agar 2216 containing peptone (1.0 g/L) and yeast extract (0.1 g/L). After the aerobic incubation of 3 days, a cream colony designated as Hm43 was selected, and the purity was confirmed by repeated streaking.

After cells were aerobically grown in 2216E medium at 30°C for 24 h with shaking at 140 rpm, genomic DNAs were extracted by using the E.Z.N.A. Bacterial DNA Kit (Omega Bio-Tek), and then were sent to Benagen Technology Co., Ltd. (Wuhan, China) for sequencing. DNA concentration and purity were assessed using a Qubit 4.0 fluorometer (Thermo Fisher Scientific) and a NanoDrop One spectrophotometer (Thermo Fisher Scientific). Illumina libraries were prepared with NEBNext Ultra DNA Library Prep Kit for Illumina (New England Biolabs, USA), with DNA fragmented to an insert size of 200–400 bp. ONT libraries were prepared using the SQK-LSK109 Sequencing Kit protocol (Oxford Nanopore Technologies), with the fragment size of >10 Kb selected by the BluePippin system. Illumina NovaSeq 6000 and Oxford Nanopore PromethION (R10.4.1 flow cell) platform sequencing generated 4,436,408 paired-end reads (2 × 150 bp) with a coverage of 353× and 95,915 reads with an N50 of 20,193 bp.

Default parameters were used, except where otherwise noted. Illumina reads were filtered with Trimmomatic v0.39, and ONT reads were filtered by quality (≥Q7) and length (≥1,600 bp) following basecalling used by Dorado v1.0. Following the assembly with Unicycler v.0.5.0 (8), gene prediction was performed using Prokka v1.14.5 (9). Carbohydrate-active enzymes (CAZymes) were annotated using the dbCAN3 webserver (https://bcb.unl.edu/dbCAN2/index.php) accessed on 25 November 2025. The 16S rRNA gene sequence identity was analyzed against the EzBioCloud webserver accessed on 8 November 2025 (10). Phylogenomic reconstruction was performed using IQ-Tree v1.6.12 (11) based on concatenated markers from GTDB-Tk v1.6.0 (12). Chitinase activity was determined as described by Hirano et al. (13) in three biological triplicates, with one unit defined as 1 μmol pNP released per minute.

**Fig 1:**
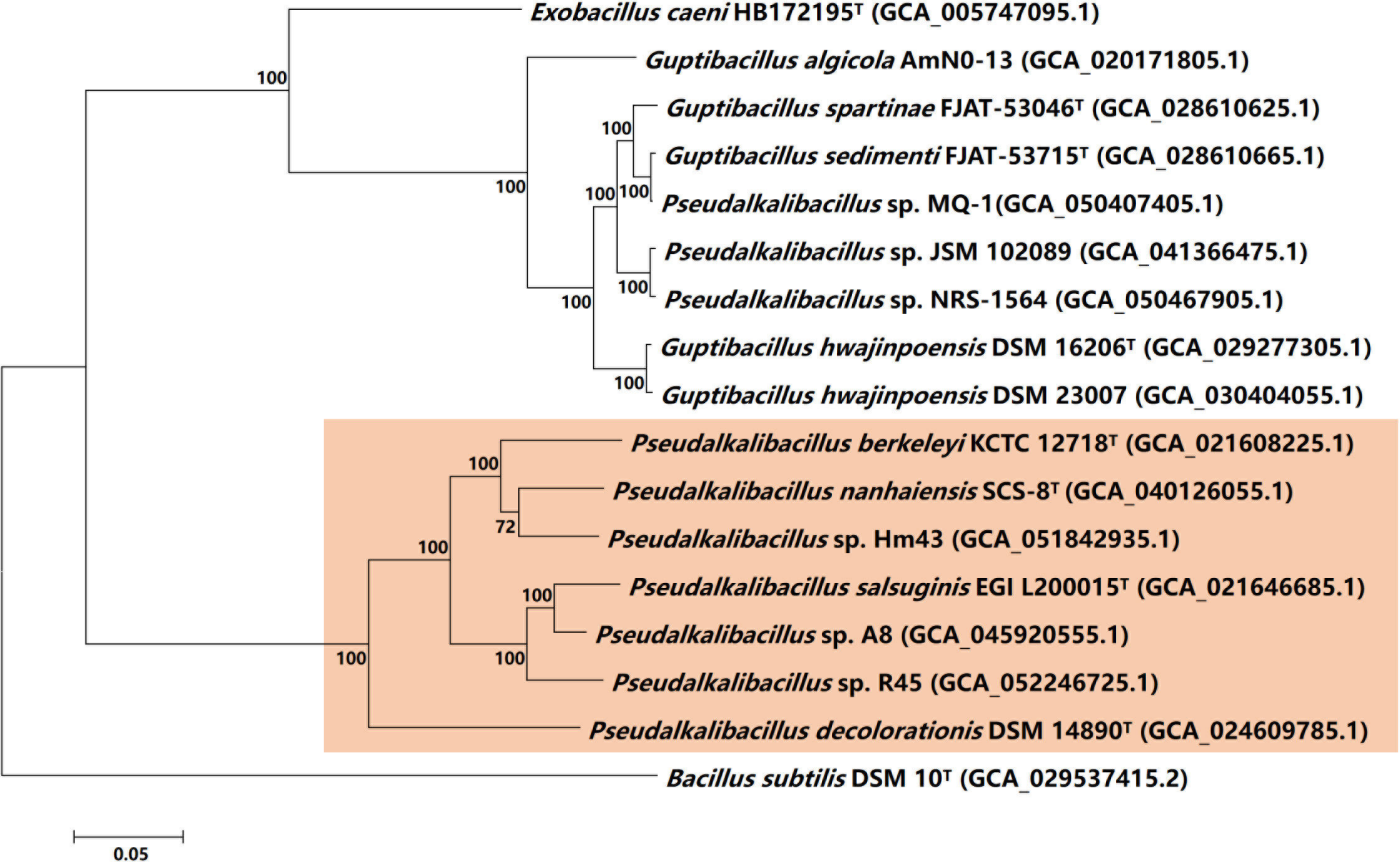
Maximum-likelihood phylogenomic tree based on the GTDB database showing the phylogenetic relationship of strain Hm43 and its related strains. The amino acid substitution model was selected as LG+F+R4 following the estimation of the best substitution model. The genus *Pseudalkalibacillus* is labeled in the orange background. Bootstrap values were based on 1,000 replicates. Bar, 0.05 substitutions per amino acid position. *Bacillus subtilis* DSM 10^T^ (GCA_029537415.2) was used as an outgroup.

The genome of strain Hm43 is a single circular chromosome of 3.74 Mbp with a guanine-cytosine (GC) content of 43.4% and contains eight rRNA operons, 74 tRNA genes, and 3,812 CDSs (Table 1). Phylogenomic analysis places strain Hm43 within the genus *Pseudalkalibacillus* (Fig. 1), with the highest 16S rRNA gene sequence identities of 97.4–99.1% with *Pseudalkalibacillus* type strains. A total of three GH18 genes (ORF_430, ORF_1383, ORF_1616) are identified as putative chitinases. Furthermore, strain Hm43 exhibits exochitinase activity of (2.8 ± 0.6) × 10^−3^ U/mL and endochitinase activity of (1.7 ± 0.5) × 10^−3^ U/mL.

**TABLE 1:** Genome annotation statistics of *Pseudalkalibacillus* sp. Hm43

Characteristic	*Pseudalkalibacillus* sp. Hm43
Total bases (bp)	3,739,586
GC content (%)	43.4
CDSs	3,812
Complete rRNAs (16S, 23S, 5S)	8, 8, 8
tRNA genes	74
CAZyme genes/families	85, 34

## Data Availability

The genome sequence of strain Hm43 has been deposited in NCBI under accession number CM135129 with BioProject and BioSample accession numbers PRJNA481858 and SAMN53055861, respectively. The raw data for the genome sequence of strain Hm43 have been deposited in NCBI SRA database under accession numbers SRR38206476 (Illumina NovaSeq 6000 platform) and SRR38206475 (Oxford Nanopore PromethION platform).
